# Research on High-Responsivity Si/Ge-APD in Visible–Near-Infrared Wide Spectrum with Light-Absorption-Enhanced Nanostructure

**DOI:** 10.3390/s25041167

**Published:** 2025-02-14

**Authors:** Guangtong Guo, Weishuai Chen, Kaifeng Zheng, Jinguang Lv, Yupeng Chen, Baixuan Zhao, Yingze Zhao, Yuxin Qin, Xuefei Wang, Dan Gao, Jingqiu Liang, Weibiao Wang

**Affiliations:** 1 Changchun Institute of Optics, Fine Mechanics and Physics, Chinese Academy of Sciences, Changchun 130033, China; 15563712841@163.com (G.G.); chenws159@163.com (W.C.); zhengkf@ciomp.ac.cn (K.Z.); lvjg@ciomp.ac.cn (J.L.); chenyp@ciomp.ac.cn (Y.C.); zhaobaixuan@ciomp.ac.cn (B.Z.); zhaoyingze@ciomp.ac.cn (Y.Z.); qinyuxindavid@163.com (Y.Q.); wangxf@ciomp.ac.cn (X.W.); 2 University of Chinese Academy of Sciences, Beijing 100049, China; 3 State Key Laboratory of Applied Optics, Changchun 130033, China; 4 Key Laboratory of Optical System Advanced Manufacturing Technology, Chinese Academy of Sciences, Changchun 130033, China; 5 School of Physics, Tonghua Normal University, Tonghua 134000, China; gx2zht@126.com

**Keywords:** Si/Ge avalanche photodiode, surface photon-trapping nanoholes, reflective grating, visible–near infrared, high responsivity

## Abstract

Photodetectors with broad spectral response and high responsivity demonstrate significant potential in optoelectronic applications. This study proposes a Si/Ge avalanche photodiode featuring nanostructures that enhance light absorption. By optimizing the device epitaxial structure and these nanostructures, a wide spectral responsivity from 0.4 to 1.6 μm is achieved. The results demonstrate that introducing surface photon-trapping nanoholes and SiO_2_ reflective grating nanostructures increases the average light absorptivity from 0.64 to 0.84 in the 0.4–1.1 μm range and from 0.31 to 0.56 in the 1.1–1.6 μm range. At an applied bias of 0.95 V_br-apd_, the responsivity reaches 17.24 A/W at 1.31 μm and 17.6 A/W at 1.55 μm. This research provides theoretical insights for designing high-responsivity photodetectors in the visible–near-infrared broadband spectrum.

## 1. Introduction

Photodetectors operating in the visible and near-infrared bands hold significant value in applications such as optical communication, spectral imaging, environmental monitoring, and biomedical diagnostics [1,2,3,4,5]. Conventional wide-spectrum detection in these bands typically relies on combining multiple discrete narrowband detectors, resulting in complex, bulky detection systems that fail to meet the increasing demand for compact, highly integrated broadband photodetectors in optoelectronic applications.

Due to the excellent light absorption properties of Ge in the telecom band and Si in the visible and short-wave near-infrared bands, as well as the compatibility of silicon and germanium epitaxial growth with silicon-based CMOS and electronic circuit manufacturing technologies [6,7], the combination of silicon and germanium enables the fabrication of low-cost, high-performance, visible-to-near-infrared (VIS-NIR) wide-spectrum photodetectors. Various high-performance detectors, fabricated from Si and Ge materials, have been validated for applications in free-space and waveguide-integrated scenarios [8,9,10,11,12]. Recent achievements including Ge/Si-based lateral waveguide photodetectors are employed for light detection in the near-infrared optical communication band [13,14,15,16,17,18,19]. Si/Ge dual-band photodetectors, with surface incidence, achieve detection in the visible or near-infrared band by adjusting the bias voltage of the two diodes [20,21,22]. Surface-normal-incidence vertical Ge/Si avalanche photodiodes (APDs), due to the excellent near-infrared light absorption of Ge and the superior avalanche multiplication characteristics of Si, have been used for high-sensitivity light detection in optical communication bands [23,24,25,26]. These studies demonstrate that photodetectors prepared by combining Si and Ge can achieve high-sensitivity detection in the near-infrared band; however, research on VIS-NIR wide-spectrum photodetectors remains limited. Due to the high light absorption coefficient and surface reflectivity of Ge in the visible spectrum, Ge/Si photodetectors currently face challenges in achieving high-efficiency detection in the visible range. Moreover, as Ge has a relatively low optical absorption coefficient in the near-infrared band, the thickness of the absorption region in Si/Ge photodetectors often requires a trade-off between the light absorption efficiency in the visible and near-infrared bands and the response bandwidth. Designing a device structure that facilitates high-efficiency detection across the VIS-NIR broadband is a critical issue that requires resolution.

In recent years, photon-trapping micro–nanostructures have demonstrated remarkable capabilities in photon management and enhanced photon–material interactions in optoelectronic devices, exhibiting enhanced light absorption through various photon capture mechanisms. Among them, surface nanostructures (nanowires, nanopores, nanopillars, etc.) have been applied to enhance light absorption in photodetectors [27,28,29,30,31,32,33]. The introduction of reflective structures such as ZnO and SiO_2_ has effectively improved the light absorption in the near-infrared band of solar cells [34,35,36,37]. To meet the demand for high-responsivity photodetection across the VIS-NIR wide spectrum, this study combines the advantages of Ge’s high-efficiency light absorption in the near-infrared band and Si’s relatively high ionization coefficient ratio for electrons and holes. A Si/Ge-APD with enhanced light absorption nanostructures is designed, achieving a wide spectral response from 0.4 to 1.6 μm. By incorporating surface photon-trapping nanoholes and SiO_2_ reflective grating nanostructures, the average light absorptivity in the 0.4–1.1 μm range is increased from 0.64 to 0.84, while in the 1.1–1.6 μm range, it is improved from 0.31 to 0.56. The responsivity at 1.31 μm and 1.55 μm reaches 17.24 A/W and 17.6 A/W, respectively. This study provides theoretical guidance for the design of VIS-NIR wide-spectrum, high-responsivity Si/Ge photodetectors, showcasing significant potential for applications in broadband detection and related fields.

## 2. Si/Ge-APD Structure Design

**Figure 1:**
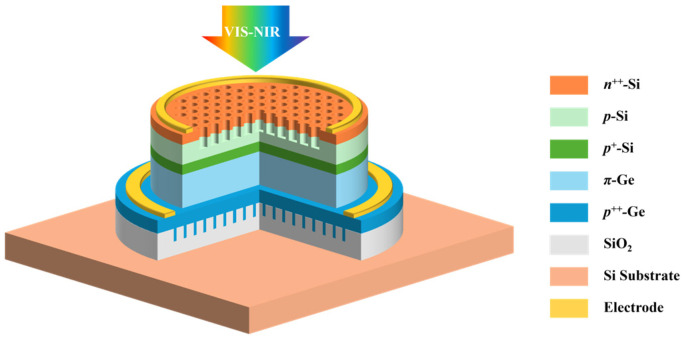
Schematic diagram of Si/Ge-APD cross-section with light-absorption-enhanced nanostructures.

Compared to Si, Ge has a higher optical absorption coefficient and shallower absorption depth in the visible light range (the absorption depth of Ge at a wavelength of 0.7 μm is approximately 0.1 μm, as shown in Figure S1 in the Supplemental Material), resulting in most photogenerated carriers recombining near the shallow surface of Ge. Consequently, the front-positioned Ge absorption layers commonly used in surface-incident Si/Ge-APDs struggle to achieve high-efficiency detection in the visible light range. To enable wide-spectrum detection in silicon–germanium photodetectors, this study designs a top-illuminated vertical Si/Ge-APD with light-absorption-enhanced nanostructures, as shown in Figure 1. Given that Si exhibits excellent light absorption properties in the visible spectrum and Ge performs well in the near-infrared range, this device structure positions the Si multiplication layer and field-control layer in the front, with the Ge absorption layer placed behind, achieving high-efficiency absorption across both visible and near-infrared spectra. Additionally, surface photon-trapping nanoholes are employed to reduce surface reflectivity and enhance detection efficiency in the visible light range, while a SiO_2_ reflective grating structure is used to boost back-reflection, thereby confining more near-infrared light within the epitaxial layer of the device, further enhancing detection efficiency in the near-infrared band.

## 3. Design and Analysis of Si-APD Epitaxial Structure

### 3.1. Parameter Design of Epitaxial Layer

To achieve high-efficiency absorption of incident light across the VIS-NIR spectrum and a lower operating voltage, the initial parameters for the device’s epitaxial structure were set as follows: the doping concentration of the n^++^-Si surface non-depleted layer is 1.0 × 10^19^ cm^−3^ with a thickness of 0.1 μm; the doping concentration of the p-Si multiplication layer is 1.0 × 10^16^ cm^−3^ with a thickness of 0.6 μm; the doping concentration of the p^+^-Si field-control layer is 1.0 × 10^17^ cm^−3^ with a thickness of 0.15 μm; the doping concentration of the π-Ge absorption layer is 1.0 × 10^15^ cm^−3^ with a thickness of 1.5 μm; the doping concentration of the p^++^-Ge electrode layer is 1.0 × 10^19^ cm^−3^ with a thickness of 0.5 μm; and the thickness of the SiO_2_ layer is 1 μm.

**Figure 2:**
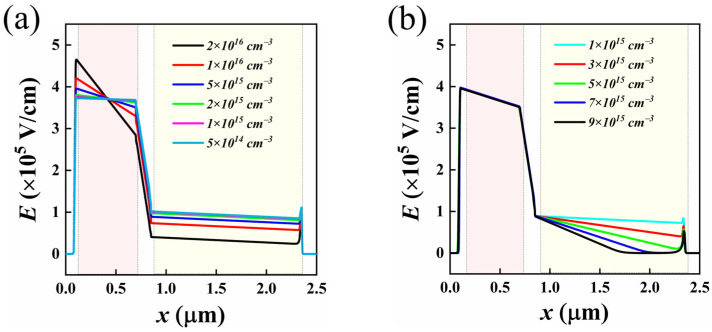
Electric field distributions within the device at different doping concentrations of the multiplication layer (**a**) and the Ge absorption layer (**b**) (the light red shaded area corresponds to the Si multiplication layer, and the light yellow shaded area corresponds to the Ge absorption layer).

In the Si/Ge-APD, the internal electric field distribution significantly influences the carrier transport behavior, thereby affecting the optoelectronic performance of the device. Based on the electric field distribution in the depletion region of the PN junction, the electric field distribution within the designed Si/Ge-APD was derived (as detailed in Supplemental Material Section S2.1). By adjusting the doping concentration and thickness of each layer, the field strength distribution within these layers can be controlled. Using the initial structural parameters set above, a two-dimensional simulation of the Si/Ge-APD was performed, keeping the parameters of other layers constant, to investigate the impact of the doping concentration of the multiplication and field-control layers on the internal electric field distribution. The multiplication layer is the main region where carriers experience impact ionization, and its doping concentration has a significant effect on the avalanche multiplication of carriers. The simulation results for the electric field distribution within the device at different doping concentrations of the multiplication layer are shown in Figure 2a. As observed, the maximum internal electric field strength increases, while the field strength in the field-control and absorption layers decreases with an increase in the doping concentration of the multiplication layer. Based on the impact ionization characteristics of Si and Ge, when the electric field strength in Si exceeds 1 × 10^4^ V·cm^−1^, or that in Ge exceeds 5 × 10^3^ V·cm^−1^, the carrier transport reaches its saturation drift velocity. When the electric field strength in Ge exceeds 1 × 10^5^ V·cm^−1^, impact ionization effects are triggered [38,39]. To achieve a high response bandwidth for the device, carriers should move at their saturation drift velocity within the device. Simultaneously, to minimize the noise current, the impact ionization effect should only occur within the multiplication layer. Therefore, the electric field strength at the interface between the Si field-control layer and the Ge absorption layer should be between 1 × 10^4^ V·cm^−1^ and 1 × 10^5^ V·cm^−1^. From Figure 2a, it can be observed that when the doping concentration of the multiplication layer exceeds 5 × 10^15^ cm^−3^, the edge field strength of the field-control layer exceeds 1 × 10^5^ V·cm^−1^. Considering this, a doping concentration of 5 × 10^15^ cm^−3^ for the multiplication layer was selected. Figure 2b shows the internal electric field distribution of the device at different doping concentrations of the Ge absorption layer. It can be seen that when the doping concentration of the Ge absorption layer exceeds 5 × 10^15^ cm^−3^, the electric field is reduced to zero before reaching the edge of the absorption layer. Taking into account the electric field distribution and breakdown voltage (V_br-apd_) of the device, a doping concentration of 5 × 10^15^ cm^−3^ for the Ge absorption layer was selected.

### 3.2. Analysis of the Photoelectric Characteristics of Si/Ge-APD

#### 3.2.1. I–V Characteristics and Avalanche Multiplication Characteristics

**Figure 3:**
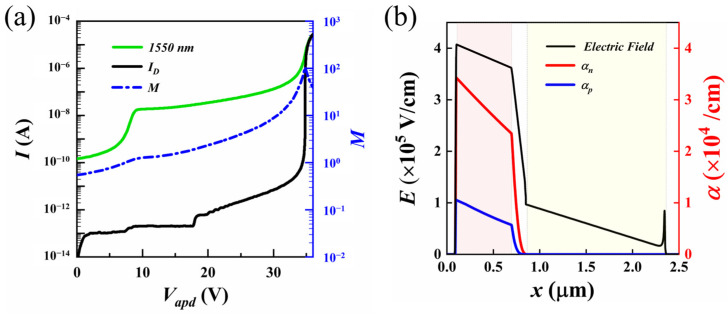
I–V characteristics and avalanche multiplication characteristics of Si/Ge-APD. (**a**) Photocurrent, dark current, and multiplication coefficient (M) curves within the Si/Ge-APD under different applied biases; (**b**) electric field distribution and carrier ionization rate distribution within the Si/Ge-APD (the light red shaded area corresponds to the Si multiplication layer, and the light yellow shaded area corresponds to the Ge absorption layer).

Based on the structural parameters mentioned above, the simulation results show the relationship between the reverse current and the applied bias voltage for the Si/Ge-APD under dark conditions, as depicted in Figure 3a. It was observed that the dark current of the Si/Ge-APD increases with the applied bias voltage, but the trend of the dark current variation differs. This phenomenon can be attributed to the differing current generation mechanisms under various applied bias voltages: When the applied bias is relatively low, the device is in a non-pull-through state, resulting in a small dark current, with the corresponding multiplication coefficient remaining nearly constant (M ≈ 1). In this state, the dark current is primarily composed of generation–recombination current and lower diffusion current. As the applied bias increases further, the device is pulled through as a whole, the width of the depletion region increases, the electric field strength in the multiplication region intensifies, and more carriers undergo avalanche multiplication in the multiplication region. These carriers are rapidly separated under the influence of the electric field in the depletion region, leading to a significant increase in the dark current and a marked rise in the multiplication coefficient. At this point, the dark current is mainly driven by the higher avalanche multiplication current and generation–recombination current. As the applied bias approaches or reaches the avalanche breakdown voltage of the device (V_br-apd_ = 34.83 V), the ionization rate of carriers increases rapidly with the growing electric field strength in the multiplication region, resulting in a sharp increase in both the dark current and the multiplication coefficient. The electric field distribution within the Si/Ge-APD at the breakdown voltage is shown in Figure 3b. It can be observed that the device is in the fully-on operating state at the point of breakdown, with the electric field strength in the Ge absorption layer exceeding 5 × 10^3^ V·cm^−1^ and the field strengths in the field-control and multiplication layers exceeding 1 × 10^4^ V·cm^−1^. This indicates that carriers can move at their saturation drift velocity throughout the entire device. The maximum field strength in the multiplication layer ranges from 3 × 10^5^ V·cm^−1^ to 7 × 10^5^ V·cm^−1^, suggesting that avalanche breakdown effects occur only in the multiplication layer. From the ionization rate curve in Figure 3b, it is evident that both electrons and holes experience significant ionization in the multiplication layer, indicating that carrier multiplication primarily occurs through impact ionization in this layer, leading to the amplification of the photocurrent. Furthermore, the ionization rate for electrons is higher than that for holes, implying that the device primarily operates through electron multiplication.

#### 3.2.2. Spectral Responsivity Characteristics

**Figure 4:**
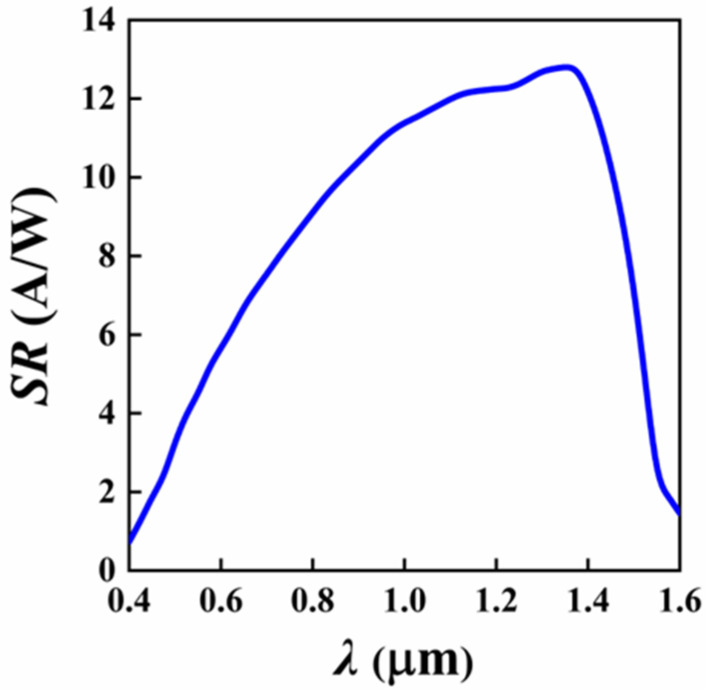
Spectral responsivity curve of the Si/Ge-APD (V_apd_ = 0.95 V_br-apd_).

The spectral responsivity (SR) characterizes the detector’s ability to convert light into an electrical signal and is defined as the ratio of the photocurrent to the incident optical power. The SR of the Si/Ge-APD in the 0.4–1.6 μm range was calculated when the device was biased at 0.95 V_br-apd_, and the results are shown in Figure 4. As shown in the figure, the designed Si/Ge-APD exhibits a wide spectral response from 0.4 to 1.6 μm, with a peak responsivity at a wavelength of 1.35 μm, where the corresponding responsivity is 12.8 A/W. The figure also shows that the responsivity is relatively low in the 0.4–0.6 μm wavelength range. This phenomenon can be attributed to two factors: first, the high surface reflectivity of the device in this range, and second, the shallow absorption depth of the device for incident light in this band, which causes most of the photogenerated carriers to recombine near the shallow surface, and carriers entering the multiplication layer do not gain sufficient energy to produce significant impact ionization effects. When the incident light wavelength exceeds 1.4 μm, the responsivity of the device rapidly decreases with increasing wavelength. This is due to the relatively thin 1.5 μm Ge absorption layer, designed to optimize high-speed response characteristics. According to the relationship between the absorption depth of Ge and the incident light wavelength shown in Figure S1b (Supplemental Material), only a small portion of the incident light is absorbed in the Ge absorption layer, while most of the light is transmitted directly into the substrate layer, resulting in fewer photogenerated carriers.

## 4. Design and Analysis of APD Light-Absorption-Enhancement Nanostructure

Based on the device’s working principle and the absorption characteristics of incident light at different wavelengths, light-absorption-enhanced nanostructures, including surface photon-trapping nanoholes and a SiO_2_ reflective grating, were designed to enhance the device’s light absorption across the VIS-NIR wide spectrum.

**Figure 5:**
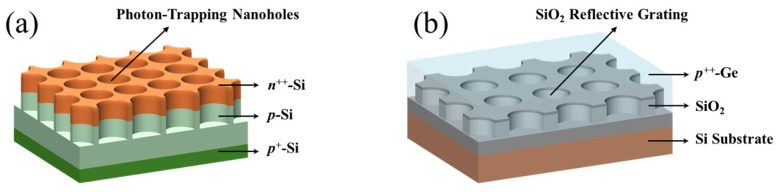
Schematic diagram of APD light-absorption-enhancement nanostructure. (**a**) Schematic of the surface photon-trapping nanoholes; (**b**) schematic of the SiO_2_ reflective grating (the surface photon-trapping nanoholes are arranged in a square lattice, with air filling the holes; the SiO_2_ reflective grating consists of periodic square-arranged SiO_2_ circular holes, with Ge filling the holes).

The epitaxial layer has a high surface reflectivity and a shallow absorption depth for visible light. As a result, most of the incident visible light is either reflected at the surface or absorbed by the surface non-depleted layer, with only a small fraction of photons reaching the depletion region to generate photogenerated carriers. To address this, surface photon-trapping nanoholes were designed. By adjusting the filling factor of the air in the nanoholes, the refractive index of the surface non-depleted layer and the multiplication layer can be modulated to reduce light reflection. Moreover, the interaction between light and the surface nanoholes allows the incident light to penetrate deeper into the depletion region, where it is absorbed to generate more photogenerated carriers, thereby enhancing the responsivity. For near-infrared light, which has high transmittance through the epitaxial layer, a SiO_2_ reflective grating is placed at the bottom of the epitaxial layer. This structure effectively confines most of the near-infrared light within the epitaxial layer, thereby increasing the light absorption efficiency of the device in the near-infrared band. Figure 5a,b illustrate the schematic diagrams of the surface photon-trapping nanoholes and SiO_2_ reflective grating structures, respectively. The structural parameters of the surface photon-trapping nanoholes are as follows: a period of 400 nm, a radius of 180 nm, and a height of 400 nm. The SiO_2_ reflective grating has a period of 800 nm, a radius of 340 nm, and a height of 500 nm. The optimization process for these parameters is detailed in Figures S2 and S3 (Supplemental Material).

**Figure 6:**
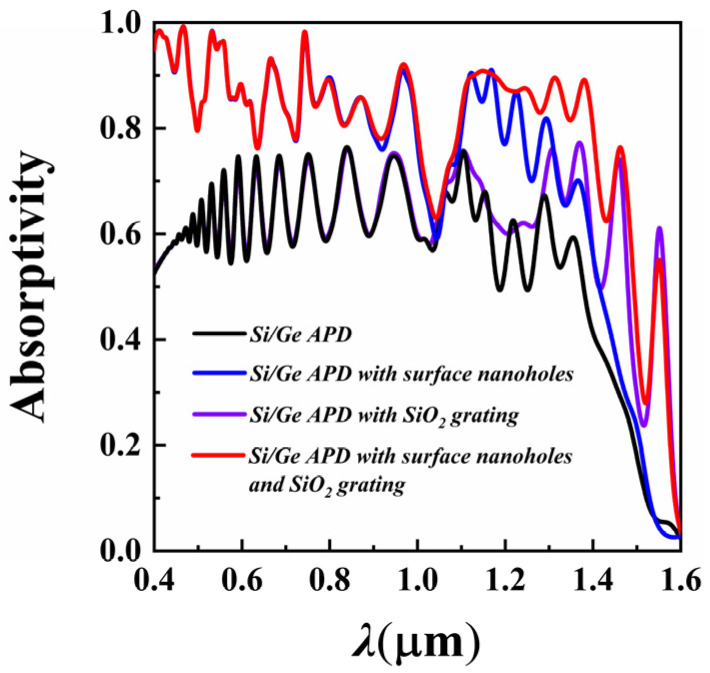
Comparison of light absorptivity of the Si/Ge-APD with and without nanostructures (the black curve represents the optical absorptivity of the APD without nanostructures, the blue curve represents the light absorptivity of the APD with only surface photon-trapping nanoholes, and the red curve represents the light absorptivity of the APD with both surface photon-trapping nanoholes and the SiO_2_ reflective grating structure).

Using the FDTD numerical algorithm, the light absorption efficiency curves of the Si/Ge-APD with and without the light-absorption-enhancing structures in the 0.4 to 1.6 μm wavelength range were calculated, as shown in Figure 6. It can be observed that introducing the photon-trapping nanoholes at the surface of the APD significantly enhances the device’s light absorptivity across the VIS-NIR spectrum. Specifically, the average light absorptivity in the 0.4–1.1 μm range increases from 0.64 to 0.84, while in the 1.1–1.6 μm range, the average light absorptivity increases from 0.31 to 0.40. Furthermore, it can be seen that after the introduction of the SiO_2_ reflective grating, the light absorptivity in the 0.4–1.1 μm range remains almost unchanged. However, the average light absorptivity increases from 0.40 to 0.56 in the 1.1–1.6 μm range. Notably, the device’s light absorptivity at 1.31 μm increases from 0.76 to 0.91, and at 1.55 μm, it rises from 0.04 to 0.53.

**Figure 7:**
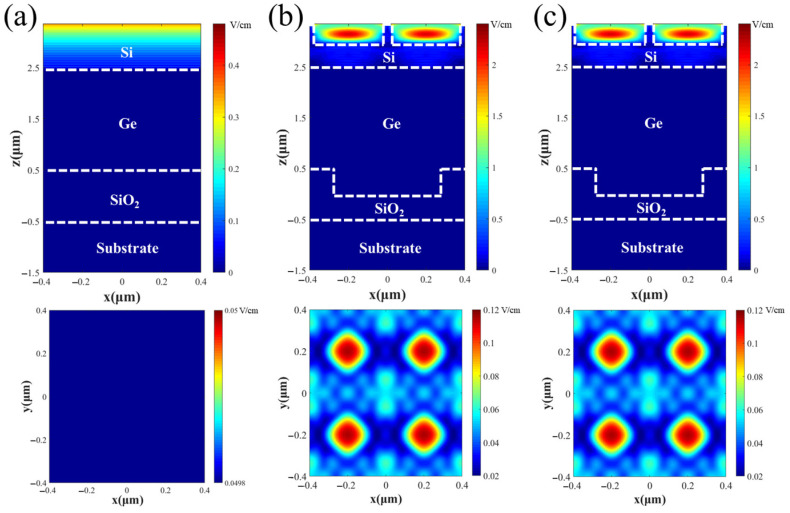
Cross-sectional view of the internal electric field strength distribution within the Si/Ge-APD with and without nanostructures at an incident light wavelength of 0.45 μm: (**a**) without nanostructures; (**b**) with only surface nanoholes; (**c**) with both surface nanoholes and the SiO_2_ reflective grating structures. (The electric field strength of the incident light is E = 1 V/m. The top row of pictures shows the electric field strength distribution in the xz cross-section, and the bottom row of pictures shows the electric field strength distribution in the xy cross-section at the interface of Si and Ge).

To investigate the mechanism by which the surface photon-trapping nanoholes and SiO_2_ reflective grating structures enhance light absorption in the Si/Ge-APD over the VIS-NIR range, the electric field strength of the incident wave and the electric field strength distributions of incident light inside the Si/Ge-APD were calculated by the FDTD numerical algorithm using 0.45 μm and 1.55 μm incident light as an example. The electric field strength distribution inside the Si/Ge-APD with and without nanostructures at an incident light wavelength of 0.45 μm was calculated and is shown in Figure 7. From Figure 7a, it can be seen that without the light-absorption-enhancing surface nanoholes, the electric field distribution is primarily concentrated near the shallow surface of the Si, and the field strength is relatively low. This is because most of the incident light is reflected at the surface, and the light absorption depth of Si at 0.45 μm is small. From Figure 7b,c, it can be observed that the electric field distribution is mainly concentrated at the bottom of the nanoholes, and the field strength in Si is significantly enhanced compared to the case without the surface nanoholes. This is due to the fact that the introduction of the surface nanoholes suppresses the surface optical reflection of the device while enhancing the trapping of incident light in the device, so that more of the incident light is absorbed at the deeper part of the device into the Si and Ge. Additionally, from Figure 7b,c, it can be seen that the electric field distribution inside the device is almost identical, regardless of the presence of the SiO_2_ reflective grating structure. This is because the light absorption depth of Si at 0.45 μm is small, and most of the incident light is fully absorbed within the epitaxial layer before reaching the SiO_2_ layer.

**Figure 8:**
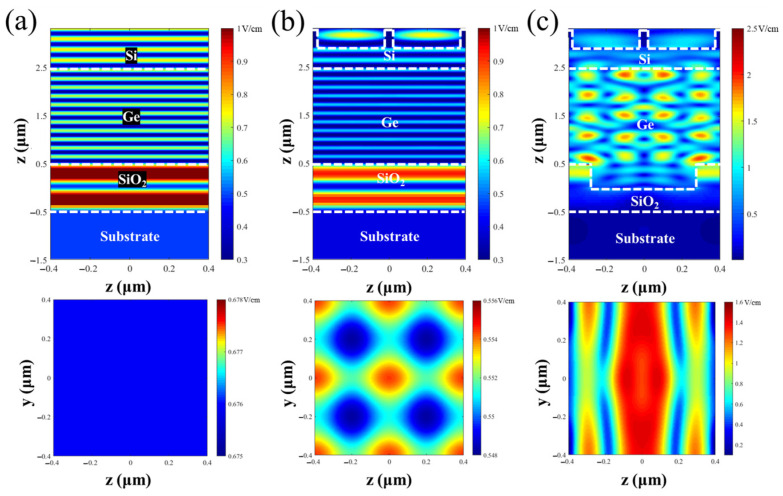
Cross-sectional view of the internal electric field strength distribution within the Si/Ge-APD with and without nanostructures at an incident light wavelength of 1.55 μm: (**a**) without nanostructures; (**b**) with only surface nanoholes; (**c**) with both surface nanoholes and the SiO_2_ reflective grating structures.

The electric field strength distribution inside the Si/Ge-APD with and without nanostructures at an incident light wavelength of 1.55 μm was calculated and is shown in Figure 8. From Figure 8a,b, it can be seen that in devices without the SiO_2_ reflective grating structure, after entering the device, the electric field gradually decays within the Si and Ge and then passes through the SiO_2_ layer into the substrate. The field strength inside the Ge, Si, and SiO_2_ layers shows a periodic enhancement distribution. This phenomenon is due to the interference effects between the incident light and the reflected light from the SiO_2_ layer, leading to periodic field strength enhancement. This periodicity is determined by the wavelength of the incident light and the refractive indices of the materials. Furthermore, compared to that for the Si/Ge-APD without nanostructures, the electric field strength in the Ge absorption layer is lower after the introduction of the surface nanoholes. This is mainly because the reverse extraction efficiency of the surface nanoholes at 1.55 μm is higher. As a result, a significant amount of reflected light is emitted through the surface nanoholes, reducing the corresponding light absorptivity (as shown in Figure 6). A detailed discussion is provided in Figure S4 (Supplemental Material). In Figure 8c, it can be observed that for the device with the SiO_2_ reflective grating structure, the field distribution is mainly concentrated in the Ge absorption layer, where the electric field is significantly enhanced at all points within the Ge layer. The maximum increase in the field strength is more than twice that of the device without the SiO_2_ reflective grating structure. This enhancement is due to the introduction of the low-refractive-index SiO_2_ reflective grating, which significantly increases the back-reflection of the incident light. Additionally, the lateral periodic SiO_2_ structure induces backward scattering, further increasing the absorption length of the Ge for incident light. Furthermore, the waveguide structure formed by the low-refractive-index SiO_2_ grating, the device epitaxial layer, and the overlying air layer traps more incident light in the Ge layer, enhancing its absorption.

**Figure 9:**
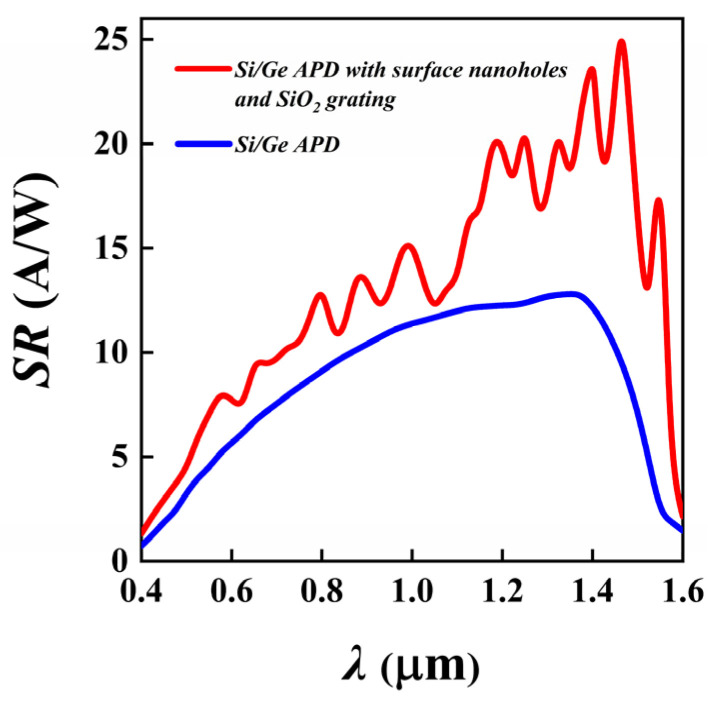
Spectral responsivity curve of the Si/Ge-APD with and without nanostructures (V_apd_ = 0.95 V_br-apd_).

Figure 9 shows the spectral responsivity curves of the Si/Ge-APD with and without nanostructures. It is evident that the introduction of surface photon-trapping nanoholes and SiO_2_ reflective grating structures significantly enhances the device’s spectral responsivity across the 0.4–1.6 μm wide spectral range, with a particularly notable improvement in the responsivity for wavelengths greater than 1.1 μm. For example, compared to that for the device without nanostructures, the responsivity at 1.31 μm increases from 12.72 A/W to 17.24 A/W, and at 1.55 μm, it rises from 2.29 A/W to 17.6 A/W. The peak responsivity in the visible light range reaches 10.89 A/W at 0.76 μm, while in the near-infrared range, the peak responsivity reaches 25.01 A/W at 1.46 μm. A brief comparison of the key parameters with other reported top-illuminated Si-Ge PDs is shown in Table 1; it shows that our designed device has high photoresponsivity across the VIS-NIR broad spectrum, indicating that the epitaxial structure of the device in this paper, as well as the light-absorption-enhancing nanostructures, is advantageous in realizing high photoresponsivity in a wide spectral band. The device has great potential for VIS-NIR broadband detection applications.

## 5. Conclusions

To meet the demand for high-responsivity light detection across the VIS-NIR wide spectral range, this study designed a Si/Ge-APD with a Si multiplication layer and field-control layer positioned at the front and a Ge absorption layer placed at the back, incorporating light-absorption-enhancing nanostructures. The basic epitaxial structure of the Si/Ge-APD, optimized for a wide spectral response, was designed to achieve a broad spectral light response from 0.4 to 1.6 μm. Additionally, based on the absorption characteristics of incident light at different wavelengths for the designed epitaxial structure, surface photon-trapping nanoholes and SiO_2_ reflective grating structures were incorporated to enhance the device’s responsivity in the VIS-NIR wide spectrum. The analysis results show that the APD with light-absorption-enhancing structures achieves an average light absorptivity of 0.84 in the 0.4–1.1 μm wavelength range and 0.56 in the 1.1–1.6 μm range. When an external bias of 0.95 V_br-apd_ is applied, the responsivity reaches 17.24 A/W at 1.31 μm and 17.6 A/W at 1.55 μm. This study indicates that the designed Si/Ge-APD, with optimized epitaxial structures and light-absorption-enhancing nanostructures, achieves wide-spectrum high responsivity in a single device, demonstrating significant potential for applications in wideband detection and other fields. This research provides theoretical guidance for the design of visible-to-near-infrared wide-spectrum, high-responsivity photodetectors.

## Figures and Tables

**Table 1:** Performance comparison of reported top-illuminated Si-Ge PDs.

**Reference**	**Device Type**	**Device Structure**	**Responsivity** **(A/W)**	**Response Band (μm)**	**Peak Response** **(A/W)**	**Dark Current (μA)**	**V_BR_** **(V)**	**Multiplication Coefficient**
[24] ^S^	APD	p^+^(Ge)-i(Ge)-p(Si)-i(Si)-n^+^(Si)	14.7 at 1.55 μm (@−19.2 V)	1.3–1.7	14.7 (1.55 μm)	7.85 (@−19.2 V)	−20.2	18.4 (@−19.2 V)
[25]	APD	p^+^(Ge)-i(Ge)-i(Si)-p(Si)-i(Si)-n^+^(Si)	19.5 at 1.55 μm (@−26.8 V)	/	/	78 (@−26.8 V)	−26.8	55.7 (@−26.8 V)
[26] ^S^	APD	p^+^(Ge)-p(Ge)-p(Si)-n(Si)-n^+^(Si)	60 * at 1.31 μm (@−26 V)	0.4–1.6	54 * (0.9 μm,@−26 V)	/	−26	60 * (@−26 V)
[40]	APD	p^+^(Si)-i(Ge)-p(Si)-n(Si)	12 at 1.55 μm (@−29 V)	/	/	5 * (@−27 V)	−29.4	39 * (@−29 V)
[41]	APD	p^+^(Si)-i(Ge)-p(Si)-i(Si)-n(Si)	8.7 at 1.55 μm (@−21 V)	/	/	3 (@−27 V)	−28.5	12 (@−27 V)
[31]	APD with photon trap	p^+^(Ge)-i(Ge)-p(Si)-i(Si)-n^+^(Si)	4.5 at 1.55 μm (@−16 V)	/	/	1 * (@−4 V)	−16	20 * (@−16 V)
[42]	APD with photon trap	n^+^(Ge)-i(Ge)-p^+^(Ge)-p^+^(Si)	0.6 at 1.55 μm (@−0.1 V)	/	/	100 * (@−8.6 V)	−8.8	14 * (@−8.8 V)
[43]	Photodiode	p(Ge)-i(Si)-n^+^(Si)	0.18 at 1.55 μm (@−1 V)	/	/	0.058 (@−1 V)		
[44]	Photodiode	p^+^(Si)-i(Si)-i(Ge)-n^+^(Si)	0.27 * at 1.55 μm (@−0 V)	0.4–1.6	0.62 * (1 μm, @0 V)	130 (@−2 V)		
[29] ^S^	Photodiode with photon trap	n^+^(Si)-p^+^(SiGe)-n^−^(Si)-n^+^(Si)	27.02 at 0.85 μm (@−3 V)	0.6–1.0	/	2.6 × 10^−7^ (@−2 V)		
[32]	Photodiode with photon trap	p^+^(Ge)-i(Ge)-n^+^(Si)	0.91 at 1.55 μm (@−1 V)	1.2–1.7	0.98 * (1.48 μm, @0 V)	1.0 * (@−1 V)		
This Work	APD with photon trap and reflection grating	n^++^(Si)-p(Si)-p^+^(Si)-π(Ge)-p^++^(Ge)	2.9 at 0.45 μm; 10.9 at 0.85 μm; 17.6 at 1.55 μm (@−33.1 V)	0.4–1.6	25 (1.46 μm, @−33.1 V)	2.1 × 10^−5^ (@−33.1 V)	−34.83	104 (@−34.83 V)

“/” represents data not given in the paper; “*” represents data not directly given in the paper but inferred from the original paper; “@xxxV” represents the value of the external bias voltage corresponding to the data. The superscript “^S^” represents theoretical simulation results in the references.

## Data Availability

Data underlying the results presented in this paper are not publicly available at this time but may be obtained from the authors upon reasonable request.

## Supplementary Materials

The following supporting material can be downloaded at: https://www.mdpi.com/article/10.3390/s25041167/s1, Section S1: Analysis of Light Absorption Characteristics of Ge and Si; Section S2. Si/Ge-APD Photoelectric Characterization Calculation; Section S3. Optimization of Nanostructure Parameters for Light Absorption Enhancement in Devices; Section S4. Electric Field Strength Distribution in Si/Ge-APD. Figure S1. (a) Absorption coefficients of Si and Ge at different incident wavelengths; (b) Absorption depths of Si and Ge at different incident wavelengths. Figure S2. Reflectance of devices with varying surface nanohole periods (a) and diameter (b) parameters under 0.4–1.1 μm incident light. Comparison of surface reflectance between devices with planar surface and those with optimized nanoholes surfaces (c). Figure S3. Optical absorptivity of devices with varying SiO_2_ grating periods (a), radii (b), and heights (c) parameters under 1.1–1.7 μm incident light. Figure S4. Electric field strength distributions of 1.55 μm incident light at different transmission time points inside the Si/Ge-APD without nanostructures (a), only surface light-trapping nanoholes (b), and both surface light-trapping nanohole as well as bottom surface SiO_2_ reflective grating structure (c). Figure S5. Spectral responsivity curve of the Si/Ge-APD. References in the Supplementary Material are listed in the main text references [38,45,46,47].
