# Supplementary material for: Research on High-Responsivity Si/Ge-APD in Visible–Near-Infrared Wide Spectrum with Light-Absorption-Enhanced Nanostructure
Source: Sensors (Basel). 2025 Feb 14;25(4):1167. doi: 10.3390/s25041167 (PMC11858985; doi:10.3390/s25041167)
Supplement: Supplementary file 1 [file sensors-25-01167-s001.zip › sensors-3441670-supplementary.pdf]

sensors

SUPPLEMENTAL MATERIAL

Research on high-responsivity Si/Ge-APD in the visible-near infrared wide spectrum with light absorption enhanced nanostructure

Guangtong Guo [1,2,3,4], Weishuai Chen [1,2,3,4], Kaifeng Zheng [1,3,4], Jinguang Lv [1,3,4], Yupeng Chen [1,3,4], Baixuan Zhao [1,3,4], Yingze Zhao [1,3,4], Yuxin Qin [1,3,4], Xuefei Wang [1,2,3,4], Dan Gao [5], Jingqiu Liang [1,2,3,4,*] and Weibiao Wang [1,2,3,4,*]

Section S1. Analysis of Light Absorption Characteristics of Ge and Si

The optical absorption coefficients α represents the absorption properties of semiconductor materials for photons, and when the incident light strength decays to 1/e of the initial light strength, the absorption depth of light of the corresponding wavelength within the semiconductor is d, where $d = 1/\alpha$. Based on Palik's material parameters[1], the optical absorption coefficient curves and absorption depth curves of Si and Ge in the wavelength range of 0.3 ~ 1.8 μm are calculated, as shown in Figure S1. From the figure, it can be seen that the absorption coefficients of Si and Ge decrease gradually with the increase of incident wavelength, and the absorption depth increases gradually. The absorption coefficient of Si in the near-infrared band is very small, and it cannot absorb incident light with a wavelength greater than 1.1 μm, and the absorption depth of Ge in the visible band is very small (such as the absorption depth of Ge at a wavelength of 0.7 μm is about 0.1 μm), and most of the photogenerated carriers are complexed on the shallow surface of germanium.

In order to enhance optical absorption efficiency of infrared light by the silicon-germanium photodetector as well as to reduce the recombination of photogenerated carriers on the surface layer of the device, it is therefore advantageous in device design to use silicon near the incident surface for visible light absorption and select germanium internally for near-infrared light absorption.

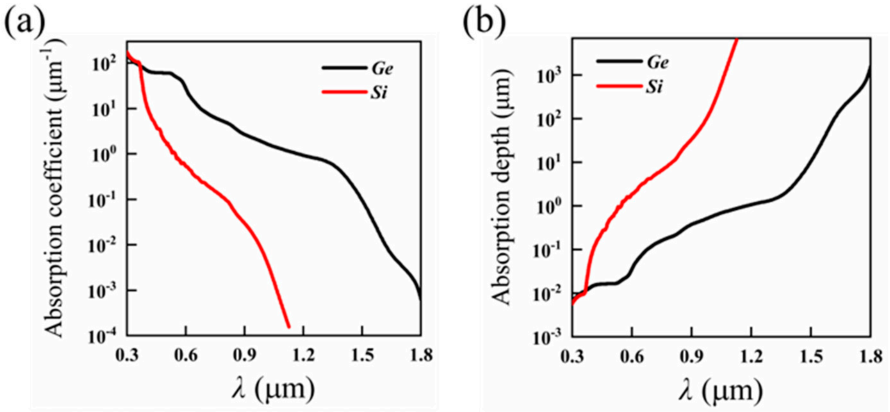

Figure S1. (**a**) Absorption coefficients of Si and Ge at different incident wavelengths; (**b**) Absorption depths of Si and Ge at different incident wavelengths

Section S2. Si/Ge-APD Photoelectric Characterization Calculation

2.1 Electric Field Distribution Within the Device

For a PN abrupt junction, the electric field strength distribution within the depletion region can be expressed as[2]: $E(x) = E_m - qNx \cdot (\varepsilon_0\varepsilon)^{-1}$, where E_m is the maximum electric field strength within the PN junction, q is the elementary charge, N is the impurity concentration, ε is the relative dielectric constant of the material, and ε_0 is the vacuum dielectric constant. Based on the above equation, the expression for the internal electric field distribution of the Si/Ge-APD shown in Figure 1 in the main text can be derived as (without considering the heterojunction and the built-in potential due to the different impurity concentrations within the homojunction):

$$E(x)=\begin{cases} E_M-\frac{qN_m}{\varepsilon_0\varepsilon_{Si}}x & (0<x<x_1)\\ E_M-\frac{qN_m}{\varepsilon_0\varepsilon_{Si}}x_1-\frac{qN_c}{\varepsilon_0\varepsilon_{Si}}(x-x_1) & (x_1<x<x_2)\\ E_M-\frac{qN_m}{\varepsilon_0\varepsilon_{Si}}x_1-\frac{qN_c}{\varepsilon_0\varepsilon_{Si}}(x_2-x_1)-\frac{qN_\pi}{\varepsilon_0\varepsilon_{Ge}}(x-x_2) & (x_2<x<x_3)\\ E_M-\frac{qN_m}{\varepsilon_0\varepsilon_{Si}}x_1-\frac{qN_c}{\varepsilon_0\varepsilon_{Si}}(x_2-x_1)-\frac{qN_\pi}{\varepsilon_0\varepsilon_{Ge}}(x_3-x_2)-\frac{qN_{Ge}}{\varepsilon_0\varepsilon_{Ge}}(x-x_3) & (x_3<x<x_4) \end{cases} \quad \text{(S1)}$$

In the equation, E_M represents the maximum electric field strength in the device, while N_m, N_c, N_π and N_{Ge} denote the impurity concentrations in the multiplication layer, field control layer, absorption layer, and electrode layer, respectively. ε_{Si} and ε_{Ge} are the relative dielectric constants of silicon and germanium materials, respectively. The maximum electric field at Ge/Si-APD junction breakdown is approximated using a one-sided abrupt junction approximation, and the maximum electric field strength E_{max} at the breakdown of a Si abrupt PN junction can be expressed as:

$$E_{\max}=\frac{4\times10^5}{1-(1/3)\log_{10}(N/10^{16})} \quad \text{(S2)}$$

Here, N represents the impurity concentration on the lightly doped side of the abrupt PN junction, which in this context corresponds to the impurity concentration in the multiplication layer (N_m).

2.2 Ionization rate

The ionization rate represents the number of electron-hole pairs produced by a single carrier moving a unit distance under a strong electric field, and the effect of the electric field strength $E(x)$ on the ionization rate of a carrier can be expressed as [3]:

$$\begin{cases} \alpha_n(x)=a_n exp\left[-\left(\frac{b_n}{E(x)}\right)\right]\\ \alpha_p(x)=a_p exp\left[-\left(\frac{b_p}{E(x)}\right)\right] \end{cases} \quad \text{(S3)}$$

In the equation, α_n and α_p denote the ionization rates of electrons and holes, respectively; a_n, b_n, a_p, b_p are the experimental parameters for the ionization rates of electrons and holes (in this context, the experimental parameters[2, 3] are taken as a_n=3.8×10^6 cm^{-1}, b_n=1.75×10^6 V·cm^{-1}, a_p=2.25×10^6 cm^{-1}, b_p=3.26×10^6 V·cm^{-1}).

2.3 Multiplication coefficient

The multiplication coefficient $M(x)$ of the APD is expressed as the ratio of the output photocurrent I_M of the carriers after avalanche multiplication effect to the initial photocurrent I_0 without multiplication. Assuming that the thickness of the APD multiplication region ranges from 0 to W_m and electrons are injected from x=0, the $M(x)$ is [2]:

$$M(x)=\frac{1}{1-\int_0^{W_m}\alpha_n\, exp\left[-\int_x^{W_m}\left(\alpha_n(x)-\alpha_p(x)\right)dx'\right]dx} \quad \text{(S4)}$$

In the equation, W_m represents the thickness of the multiplication region, and α_n and α_p are the ionization rates of electrons and holes, respectively. Moreover, the gain curve can also be derived from the experimental *I-V* curve, expressed as:

$$M=\frac{I_p-I_d}{I_{p0}-I_{d0}} \quad \text{(S5)}$$

In the equation, I_p and I_d are the photocurrent and dark current at the same bias voltage, respectively, and I_{p0} and I_{d0} are the photocurrent and dark current before the onset of multiplication, respectively.

2.4 Responsivity

The photoresponsivity of Si-APD reflects the photoelectric conversion capability of the device, which is expressed as[4]:

$$SR = M \cdot \frac{I_{p0}}{P_{in}} \approx M \cdot \frac{\lambda}{1.24} \cdot \eta = M\varphi(1-R)[1-exp(-\alpha W_D)]\frac{\lambda}{1.24} \quad (S6)$$

In the equation, λ is the wavelength of the incident light, M is the multiplication coefficient, I_{p0} is the photocurrent without avalanche multiplication, η is the quantum efficiency of the device, φ is the probability of generating an electron-hole pair per absorbed photon in the silicon, W_D is the depletion region width, R is the surface reflectance of the silicon, and α is the optical absorption coefficient of the silicon.

Based on the structural parameters of the Si/Ge-APD set in the main text, the optoelectronic characteristics of the device are calculated under ideal conditions (assuming that the impurity concentration in each layer is uniformly distributed, and the effects of lattice mismatch, interfacial states, and defects of the material on the device performance are not taken into account) (subsection 2.2 of the main text). In the simulation, the diameter of the photosensitive surface of Si/Ge-APD is 100 μm, the ambient temperature is 300 K, the power density of incident light at each wavelength is 1 W/cm^2 and perpendicularly incident to the photosensitive surface of the device, the reflectivity of the device surface is taken as the natural reflectivity of the silicon. Additionally, incorporate physical models such as Auger recombination, Selberherr impact ionization, Shockley-Read-Hall recombination, and carrier mobility to enhance the accuracy of the calculation results.

Section S3. Optimization of Nanostructure Parameters for Light Absorption Enhancement in Devices

3.1 Surface light-trapping nanoholes

The initial parameters of the nanoholes on the device surface are selected as follows: period (P = 500 nm), diameter (D = 0.8P), and height (H = 400 nm). To facilitate the absorption of incident light in the multiplication layer, the surface nanohole height is kept constant while varying the period and diameter of the nanoholes. The reflectance curves of the Si/Ge-APD with the surface nanoholes within the 0.4 to 1.1 μm wavelength range are calculated, as shown in Figure S2. The parameters corresponding to the lowest average light reflectivity in the visible (0.4-0.76 μm) range were selected to determine the structural parameters of the optimized surface nanoholes as P = 400 nm, D = 360 nm, and H = 400 nm, respectively.

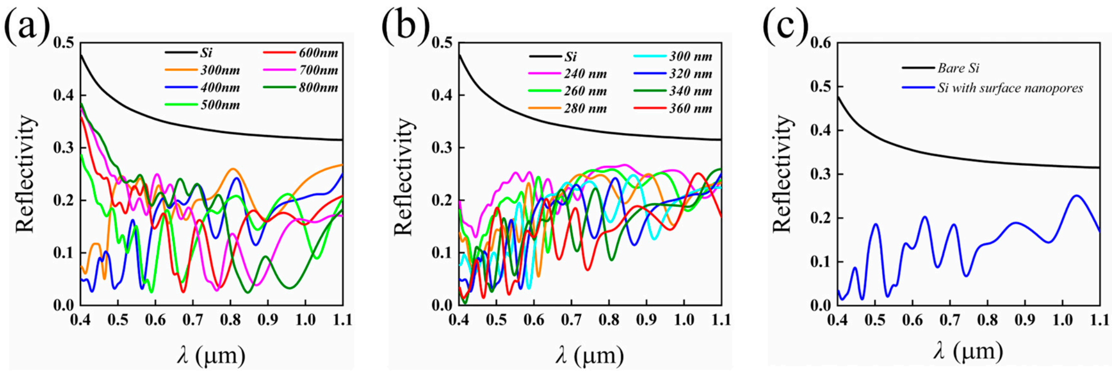

Figure S2. Reflectance of devices with varying surface nanohole periods (**a**) and diameter (**b**) parameters under 0.4-1.1 μm incident light. Comparison of surface reflectance between devices with planar surface and those with optimized nanoholes surfaces (**c**).

3.2 SiO_2 Reflective Grating Structure

A SiO_2 reflective grating structure was designed to improve the optical absorption of the device at 1.55 μm wavelength. The initial parameters of the SiO_2 reflective grating are: p=600 nm, r=0.4 p, and height h=500 nm. By varying the period and diameter of the nanoholes, the optical absorptivity curves of the Si/Ge-APD with SiO_2 reflective grating are calculated within the 0.8 to 1.7 μm wavelength range, as shown in Figure S3 (due to the transparency of Si for wavelengths greater than 1.1 μm, the design and optimization

of the SiO_2 reflective grating structure are based on the original Si/Ge-APD, excluding the surface nanoholes). The optimized SiO_2 reflective grating structural parameters are determined by selecting the parameters corresponding to the highest optical absorptivity at 1550 nm, resulting in: p = 800 nm, r = 340 nm, and h = 500 nm.

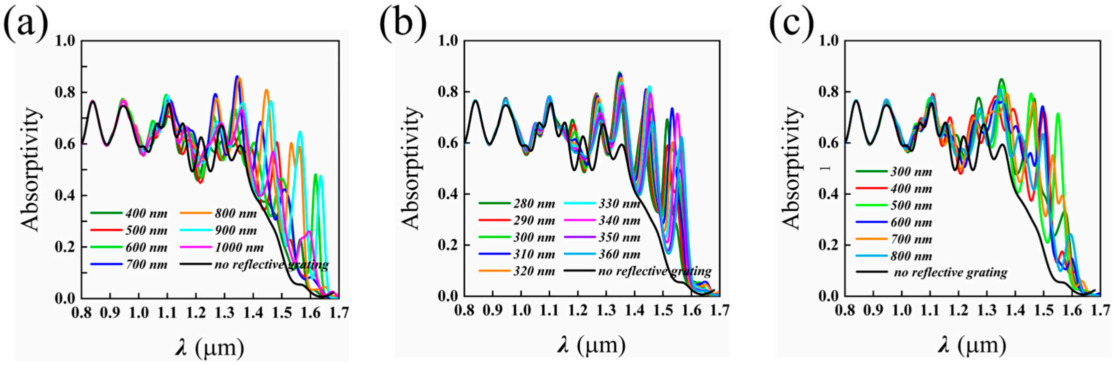

Figure S3. Optical absorptivity of devices with varying SiO_2 grating periods (**a**), radii (**b**), and heights (**c**) parameters under 1.1-1.7 μm incident light.

Section S4. Electric Field Strength Distribution in Si/Ge-APD

To elucidate the process of enhanced absorption of 1.55 μm incident light by introducing surface nanoholes and the SiO_2 reflective grating, the electric field distribution within the Si/Ge-APD at different transmission time points was calculated using the FDTD numerical algorithm, as shown in Figure S4.

Figure S4 (a) shows the electric field distribution inside the Si/Ge-APD without introducing the nanostructures, and it can be observed that the electric field strength rapidly decreases with increasing transmission time. This is due to the relatively small thickness of the epitaxial layer, where most of the incident light passes through the SiO_2 grating directly into the substrate layer or is reflected and transmitted into the air, resulting in a relatively low absorption rate of the 1.55 μm incident light in the epitaxial layer. From Figure S4(b), which illustrates the electric field distribution when only surface nanoholes are introduced, it can be observed that due to the anti-reflection properties of the surface nanoholes, more incident light enters the APD epitaxial layer (such as at 40 fs, 50 fs, and 63 fs in Figure S4(b)), but more light continues to be transmissive to the outside of the device through the surface nanoholes after the reflection from the SiO_2 layer due to the high reverse extraction efficiency of the surface nanoholes (as seen at 99, 105, 132 fs in Figure S4 (b)) , while the light entering the surface nanoholes undergoes lateral scattering, causing a portion of the light to laterally propagate within the Si layer. This results in a lower optical absorptivity at 1.55 μm compared to the Si/Ge-APD without nanostructures, as shown by the lower absorption rate at 1.55 μm in Figure 5 of the main text. As a control, As a control, the electric field distribution of 1.31 μm incident light inside the APD with only the surface nanoholes introduced was also calculated, and the results indicate that the stronger attenuation reflection and lower back-transmission that the surface nanoholes would produce for the 1.31 μm incident light resulted in the increased light absorption of the APD. Figure S4(c) displays the electric field distribution when both surface nanoholes and SiO_2 reflective grating are introduced. It shows that the SiO_2 reflective grating exhibits strong back reflection for 1.55 μm incident light, causing most of the light to be reflected back into the Ge absorption layer after passing through SiO_2, with very little direct transmission into the substrate layer (as observed at 90 fs and 112 fs in Figure S4(c)). Furthermore, the introduction of the lateral periodic grating structure induces backscattering of incident light, thereby increasing the absorption length in the Ge for incident light. This results in more light being confined and absorbed in the germanium layer (as seen at 118 fs and 145 fs in Figure S4(c)).

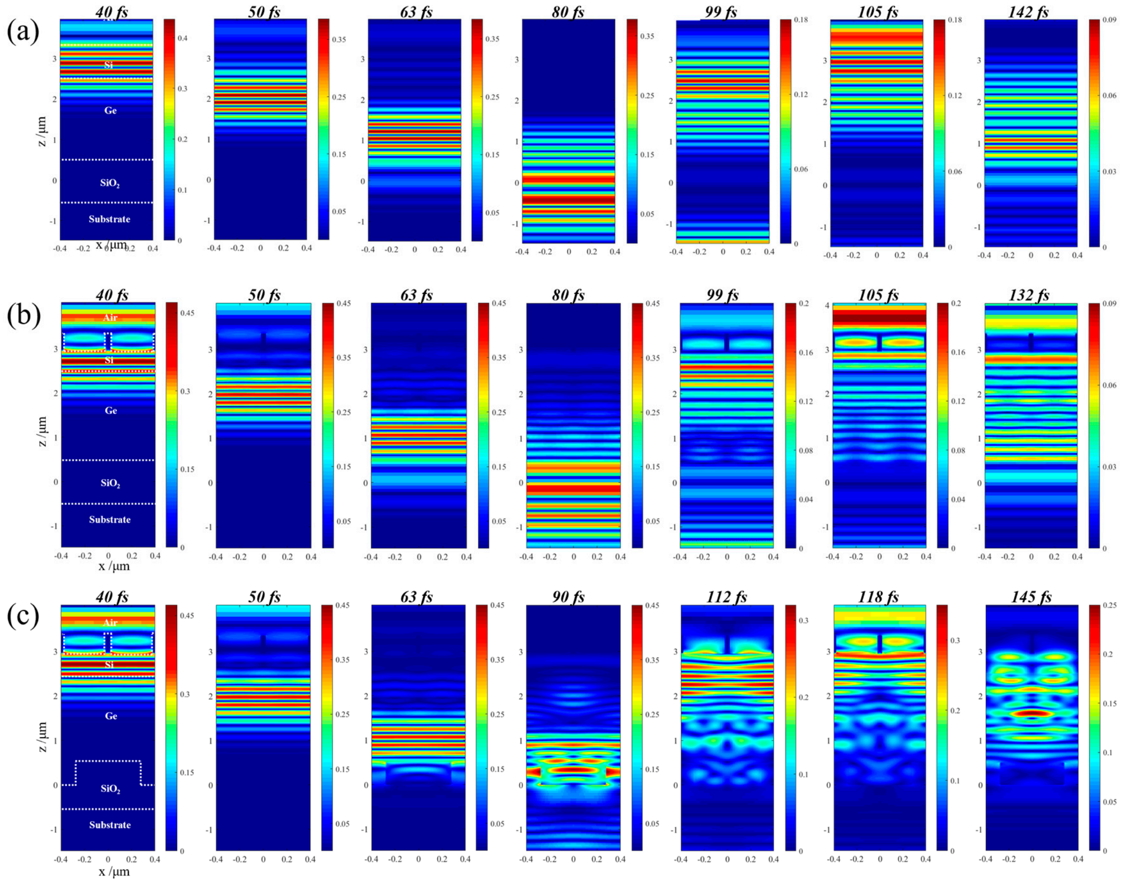

Figure S4. Electric field strength distributions of 1.55 μm incident light at different transmission time points inside the Si/Ge-APD without nanostructures (**a**), only surface light-trapping nanoholes (**b**), and both surface light-trapping nanohole as well as bottom surface SiO_2 reflective grating structure (**c**). (The figure shows the electric field strength distribution in the xz plane at y=0.2 μm, with an incident light wavelength of 1.55 μm and an electric field strength of 1 V/m; each row corresponds to specific times as the incident light enters the APD, reaches the Ge absorption layer, reaches the SiO_2 layer, undergoes gradual back reflection and transmission at the SiO_2 grating layer, with the reflected light reaching the Ge/Si interface, reaching the APD/air surface, and the remaining light re-entering the APD after surface transmission).

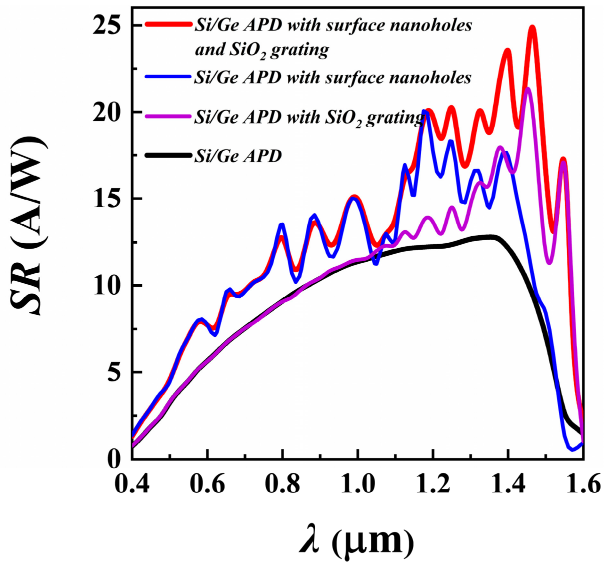

Figure S5. Spectral responsivity curve of the Si/Ge-APD

References

1. Palik, E. D. Handbook of optical constants of solids. Academic press, USA, 1985.
2. Sze, S. M.; Li, Y.; Ng, K. K. Physics of semiconductor devices, 3rd ed; Xi'an Jiaotong University Press, China, 2008.
3. Woods, M. H.; Johnson, W. C.; Lampert, M. A. Use of a Schottky barrier to measure impact ionization coefficients in semiconductors. Solid-State Electron. 1973, 16, 381–394.
4. Saleh, B. E.; Teich, M. C. Fundamentals of Photonics. John Wiley & Sons, USA, 1991.
